# Trichotillometry: the reliability and practicality of hair pluckability as a method of nutritional assessment

**DOI:** 10.1186/1475-2891-6-9

**Published:** 2007-05-01

**Authors:** Laura A Wyness, Geraldine McNeill, Gordon J Prescott

**Affiliations:** 1 Department of Public Health, University of Aberdeen, Medical School, Foresterhill, Aberdeen AB25 2ZD, UK; 2 Department of Environmental and Occupational Medicine, Liberty Safe Work Research Centre, Foresterhill Road, Aberdeen AB25 2ZP, UK

## Abstract

**Background:**

A nutritional assessment method that is quick and easy to conduct would be extremely useful in a complex emergency, where currently there is no agreed practical and acceptable method. Hair pluckability has been suggested to be a useful method of assessing protein nutritional status. The aim was to investigate the reliability of the trichotillometer and to explore the effects of patient characteristics on hair epilation force.

**Methods:**

Three observers plucked hair from twelve participants to investigate the within- and between-observer reliability. To investigate the effect of patient characteristics on hair pluckability, 12 black African and 12 white volunteers were recruited. Participants completed a short questionnaire to provide basic information on their characteristics and hair.

**Results:**

Mean hair pluckability measurements for the 12 participants obtained by the three observers (39.5 g, 41.2 g and 32.7 g) were significantly different (p < 0.001). Significant variation between patients was also found (p < 0.001). None of the patient characteristics significantly affected hair pluckability, with the exception of age, although this relationship was not consistent.

**Conclusion:**

Due to significant variation in measurements, hair pluckability does not appear to be a reliable method for assessing adult nutritional status. Hair pluckability could be a useful method of nutritional assessment in complex humanitarian emergencies but only if the reliability was improved.

## Background

Complex emergencies are usually characterised by large numbers of displaced persons and overwhelming need for shelter, water, sanitation and health care commonly due to extreme insecurity. Time and resources available during a complex emergency are often extremely limited, so a nutritional assessment method that is quick and easy to conduct is required. Mid-upper arm circumference and weight can be (and are) used in complex emergencies, although there remains some debate regarding the appropriate cut-off measurements to indicate nutritional status.

One of the signs of protein-energy malnutrition is hair that can be plucked easily [1]. Protein is the main component of hair; forming between 65 to 95% of the hair by weight [2]. During protein deficiency, blood proteins are maintained at the expense of structural protein; therefore structural proteins are an earlier indicator of protein insufficiency [3]. The hair root shows a prompt response to protein deficiency. Studies have shown that crash dieting, where the diet is severely constrained over a short period, can precipitate hair loss [4,5]. Chronic starvation, especially marasmus, is also associated with diffuse hair loss [6] and hair that is easy to pluck [7,8]. Chase *et al*. (1981) found patients with kwashiorkor or marasmic kwashiorkor had slightly decreased epilation force than patients with "pure" marasmus [9]. This should be borne in mind when assessing malnourished individuals. Further investigation of this issue was beyond the scope of this paper. In complex emergencies inexpensive and simple methods for screening for malnutrition and for assessing changes in nutritional status are required.

Two studies of measurement of hair pluckability as a possible measure of nutritional status have been reported [9,10]. In these studies, a hand-held spring dynamometer, or "trichotillometer" was used to measure the force in grams that was required to pluck individual hairs from the scalp. Chase *et al*. (1981) [9] measured the hair epilation force of 17 malnourished and 16 well-nourished hospitalized patients using the trichotillometer. Epilation force was found to be significantly correlated with weight, triceps skinfold thickness, arm muscle circumference and some laboratory assessments of nutritional status. Smelser *et al*. (1982) [10] determined the usefulness of trichotillometry under field conditions by measuring hair epilation force of 69 subjects at a hospital in Nigeria. Epilation force was found to be significantly correlated with weight-for-height, serum albumin and mid arm muscle circumference. The studies [9,10] both concluded that the trichotillometer might be a useful method for assessing protein nutritional status. However, to be useful in the field the method needs to have low within- and between-observer variation and low intra-individual variation. The present study was therefore conducted to investigate the reliability of hair pluckability as measured by a trichotillometer.

## Methods

### Study subjects

Data was collected for the survey investigating the reliability of the trichotillometer from staff and students at the University of Aberdeen. The inclusion criteria for the study were that participants were aged 18 years or over at the time of the study, and that they had hair of adequate length to be plucked, which covered the area on the top of the scalp.

Data was also collected for the comparison survey investigating the effect of participant characteristics on the epilation force required to pluck hair. The initial inclusion criteria for this study were identical to the reliability study described above. Recruitment of black African volunteers was conducted first. White volunteers were then recruited among staff and students at the University of Aberdeen and chosen to balance the black African participants according to gender and age within two years.

Sample size calculations for both proposed studies were performed using the statistical program "nQuery Advisor" (version 4.0), using data from two previous studies [9,10], to determine the required number of participants to detect a difference of 1 standard deviation (SD) assuming a power of 80%. These studies found differences in means of 21.2 g and 10.8 g between malnourished and control subjects (with SD of between 9.5 g and 11.8 g) at the 5% level of significance using unpaired t-tests [9,10]. Measurements from ten individuals were required to test the within-observer reliability and three observers were required to test the between-observer reliability. Ethical approval was obtained from Grampian Research Ethics Committee for both studies.

### Data collection and measurement technique

All participants completed a short questionnaire to provide basic information on gender, age and ethnicity. Data were also collected about the colour and length of each participant's hair, and whether the hair was currently dyed, permed or if any styling products were applied on the day hair pluckability measurements were collected.

Instructions from the developer of the trichotillometer, Prof. C.L. Krumdieck, were provided and adhered to by the observers. The trichotillometer has an arbitrary scale (units) which can be converted to grams using a calibrated scale. In this study one unit was equivalent to 2.5 g.

In both studies ten hairs from the top of each participant's head were epilated individually. Measurements typically took five minutes for ten hairs. The number of broken hairs and the number of malfunctions of the ratchet mechanism of the trichotillometer were recorded. If required additional hairs were epilated until a sample of ten hairs was achieved. Measurements obtained by three observers were used to assess the between-observer reliability. Measurements obtained from each observer were used to assess within-observer reliability.

### Statistical analysis

Analysis of the data was conducted using SPSS, version 13. A two-way ANOVA was used to investigate the variation in hair pluckability due to differences between observers and between participants. Comparisons between the measurements obtained in groups with different participant characteristics were made using the independent t-test, a one-way ANOVA or Kruskal-Wallis test as appropriate.

## Results

Of the 12 participants included in the reliability study seven were female. Seven were of white British ethnicity and five were of white, non-British ethnicity. One participant did not report her age. The mean age of the other 11 participants was 35.3 years (SD = 9.1 years). The measurements within an individual were generally normally distributed although the distributions were slightly skewed for some individuals.

### Observer variation

**Figure 1:**
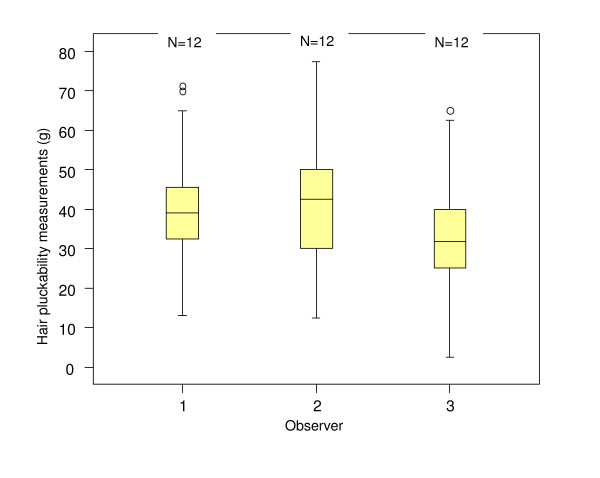
Box plot showing the distribution of hair pluckability measurements obtained across the three observers.

The differences in measurement between the three observers can be seen in Figure 1. The mean (SD) hair pluckability measurements for observer 1, 39.5 g (10.9 g), and 2, 41.4 g (13.4 g), were similar, whereas observer 3 obtained a significantly lower mean hair pluckability, 32.7 g (10.0 g). For observers 1, 2 and 3, the average ranges of individual hair pluckability measures and mean coefficients of variation (CV) were 30.5 g, 32.5 g and 28.0 g and 23.7%, 25.8% and 25.4%, respectively.

### Participant variation

**Figure 2:**
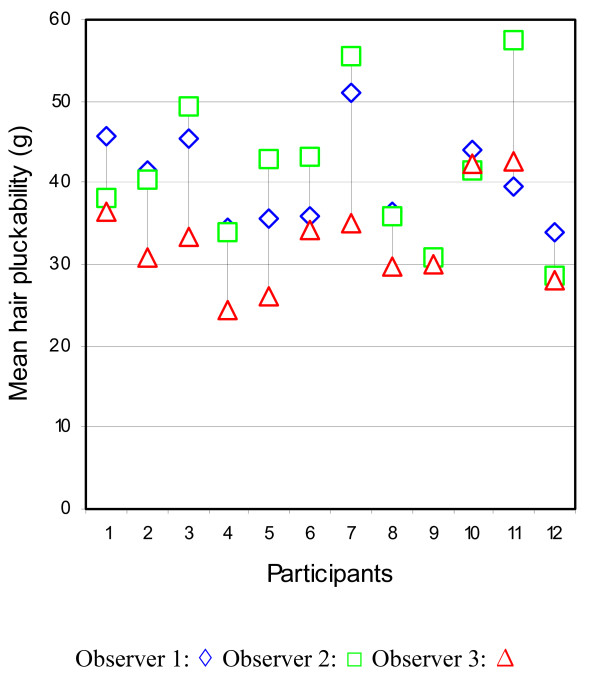
Participants mean hair pluckability measured by each observer.

Large differences in hair pluckability measurements across the 12 participants were observed (Figure 2). Some participants had similar measurements for all 10 hairs plucked.

### Two-way analysis

A two-way ANOVA was carried out to investigate the variation in hair pluckability due to observer and participant effects. While there were some individuals with skewed measurements, the residuals from this analysis were normally distributed satisfying the relevant assumptions. Variances were homogeneous in the model with an interaction effect. Significant differences in hair pluckability between observers (p < 0.001) and between participants (p < 0.001) were found as well as a significant interaction effect (p = 0.001). This indicated that the differences between individuals in terms of hair pluckability were not independent of the observer.

The effect size for the observers (partial eta squared = 0.282) and the participants (partial eta squared = 0.141) can be classified as large using Cohen's 1988 criterion [11]. Post-hoc comparisons using the Sheffe's test indicated that the mean hair pluckability for observer 3 was significantly lower than the mean obtained by observers 1 and 2 (p < 0.001). Observers 1 and 2 did not differ significantly (p = 0.261).

### Comparative study

The basic characteristics of the participants included in the comparative study and the results of the statistical analysis on the data are shown in Table 1. Hair pluckability was not significantly correlated with age. However, when participants were grouped into three age groups, a post hoc comparison using Sheffe's test indicated that the mean pluckability for the age group 30–40 years was significantly lower than the mean score for the age groups 19–29 years and 41–52 years. Therefore, there was no consistent relationship between hair pluckability and age in these data.

**Table 1:** Comparative study sample characteristics

Participant characteristic	Black African n = 12	White n = 12	Hair pluckability Mean (SD)	Test statistic (P value)
Ethnicity (number):				
Black African	12	0	39.9 (10.8)	0.89 (0.372)^1^
White Australian	0	1	38.7 (11.0)	
White British	0	9	38.7 (11.0)	
White German	0	1	38.7 (11.0)	
White Italian	0	1	38.7 (11.0)	
Gender (number)				
Male	4	4	40.9 (11.2)	1.58 (0.115)^1^
Female	8	8	38.5 (10.7)	
Age (Mean (SD))	35.3 (9.1)^2^	36.7 (9.1)	36.0 (9.1)	0.083 (0.208)^3^
Age groups				
19–29 y	4	2	41.6 (12.5)	10.38 (<0.001)^4^
30–40 y	3	6	35.4 (9.5)	
41–52 y	4	4	41.7 (10.0)	
Age groups				
19–36 y	6	6	38.4 (11.6)	1.39 (0.187)^1^
>36 y	5	6	40.2 (10.1)	
Hair color (number):				
Black	11	1	39.7 (10.9)	0.157 (0.525)^5^
Brown	0	8	38.9 (10.9)	
Blonde	0	2	38.9 (10.9)	
Grey	1	0	38.9 (10.9)	
Red	0	1	38.9 (10.9)	
Hair length (number):				
Shorter than ear length	8	8	37.5 (32.5, 45.0)^6^	1.09 (0.579)^5^
Between chin and shoulder	3	2	37.8 (29.9, 47.5)^6^	
Longer than shoulder	1	2	42.6 (29.7, 50.1)^6^	
Hair currently permed				
Yes	7	0	40.2 (10.2)	0.78 (0.436)^1^
No	4	12	38.9 (11.2)	
Hair currently dyed				
Yes	2	7	41.4 (10.5)	1.90 (0.058)^1^
No	10	4	38.4 (11.0)	
Currently have hair products on hair				
Yes	7	6	39.5 (10.9)	0.25 (0.804)^1^
No	4	6	39.1 (11.0)	
Broken hair				
≥1	3	1	41.4 (10.7)	-1.36 (0.177)^1^
None	9	11	38.9 (10.9)	

^1^Independent t-test, ^2^N = 11 as one participant did not report their age, ^3^Spearman's correlation, ^4^One-way ANOVA, ^5^Kruskal Wallis test, ^6^Median (Interquartile range)

## Discussion

The trichotillometer was previously reported to be a good method for assessing nutritional status in adults [9,10]. Although the effect of acute stress on hair pluckability using the trichotillometer [9] and the usefulness of the trichotillometer under field conditions [10] have been previously investigated, the reliability and the effect of various individuals' characteristics on hair pluckability have not been investigated. The observations presented here are consistent with Chase *et al*. [9] who found the average observed mean hair pluckability (SD) among well-nourished patients, when plucking ten individual hairs was 36.0 g (12.4 g).

Variation between observers is not desirable since similar measurements should be obtained by all observers who use the same instrument on the same individual. This study found two of the observers reported similar results and one reported significantly lower measurements by, on average 8.7 g, which in clinical terms is quite a large difference. It is possible that a variation of this magnitude would effect the classification of the nutritional status of an individual.

Variation within observers is also not desirable and, from a clinical perspective, this mean variation of close to 30 g within an observer is large. This wide variation in individual hair pluckability measurements indicates that an adequate amount of individual hairs need to be plucked in order to obtain a reproducible and representative mean hair pluckability measurement.

Variation between participants for reasons unrelated to nutritional status would not be a welcome finding, as this could obscure differences between individuals with different nutritional status. This study found significant differences between the participant's hair pluckability measurements. There was also wide variation in the ten measurements obtained for each participant as indicated by relatively large coefficient of variation (CV). A CV of >30% is often considered as large experimental variability [12]. This may be due to biological variation in hair pluckability as the force required to pluck hair is likely to vary throughout the different stages of growth. A balance between the time available and the number of hairs required to be plucked in order to obtain a valid measurement needs to be found.

A significant interaction effect was found, indicating that the differences between individuals in terms of hair pluckability were not independent of the observer. This is not desirable since the tool should be able to discriminate between individuals regardless of the observer.

No significant difference in hair pluckability between black African and white adults or between males and females was found. However, since the number of male participants was small (n = 8), a larger study may be required to ensure the results are generalisable to a wider population. There was no consistent relationship between hair pluckability and age. One result suggested that younger and older age groups required a larger force to pluck hair than the middle age group of 30–40 year olds. A larger study would be required to verify this finding due to the relatively small number of participants in this study. No significant difference in hair pluckability was identified with hair colour or length, even when the categories were combined to increase the number of participants in the smaller groups.

All three observers were given the same instructions on how to use the trichotillometer, and were allowed to practice obtaining measurements until they felt confident using it. Due to the availability of the observers and participants, observers may have taken measurements on a participant on different days. However, all measurements were obtained from all three observers within three days of each other. This may be a source of variation as the participant may have used hair products for one measurement and not another. However, it is unlikely to have had an effect as no significant association was found between hair pluckability and the use of hair products. Although none of the participants in this study objected to having ten hairs pulled out, the acceptability to some individuals may be lower, for example due to a lower pain threshold or traditional beliefs not permitting the removal of hair, as in some African cultures.

## Conclusion

In conclusion, one classification scale can be used when assessing hair pluckability for all adults as no significant differences were identified with any of characteristics of the participants. However, the differences found between the measurements obtained by each observer and wide variations in measurements within observers suggest that the trichotillometer is not a reliable method for assessing adult nutritional status. If the reliability of the trichotillometer could be improved, for example, by adjusting the design, hair pluckability would be an extremely useful method of nutritional assessment during a complex emergency, or a situation where savings on time and resources need to be made.

## Competing interests

The author(s) declare that they have no competing interests.

## Authors' contributions

LAW collected and analysed the data and wrote the manuscript. GM provided advice on the study design and collection of the data, and critically revised the manuscript. GJP provided advice on the collection and analysis of the data, and critically revised the manuscript. All authors read and approved the final manuscript.
